# MNK1 and MNK2 mediate adverse effects of high-fat feeding in distinct ways

**DOI:** 10.1038/srep23476

**Published:** 2016-04-18

**Authors:** C. E. J. Moore, J. Pickford, F. R. Cagampang, R. L. Stead, S. Tian, X. Zhao, X. Tang, C. D. Byrne, C. G. Proud

**Affiliations:** 1 Centre for Biological Sciences, University of Southampton, Southampton, SO17 1BJ, United Kingdom; 2 Nutrition and Metabolism, South Australian Health & Medical Research Institute, North Terrace, Adelaide, SA5000, Australia; 3 Institute of Developmental Sciences, University of Southampton, Faculty of Medicine, Southampton SO16 6YD, United Kingdom; 4 Department of Biochemistry & Genetics, Zhejiang University School of Medicine, Hangzhou, Zhejiang 310058 China; 5 Nutrition and Metabolism, University of Southampton, Faculty of Medicine, Southampton SO16 6YD, United Kingdom; 6 Southampton National Institute for Health Research, Biomedical Research Centre, University Hospital, Southampton, UK; 7 School of Biological Sciences, University of Adelaide, Adelaide SA 5005, Australia.

## Abstract

The MAP kinase-interacting kinases (MNK1 and MNK2) are non-essential enzymes which are activated by MAP kinases. They are implicated in controlling protein synthesis. Here we show that mice in which the expression of either MNK1 or MNK2 has been knocked out (KO) are protected against adverse effects of high-fat feeding, and in distinct ways. High-fat diet (HFD)-fed MNK2-KO show less weight gain than wild-type animals, and improved glucose tolerance, better insulin sensitivity and markedly diminished adipose tissue inflammation. This suggests MNK2 plays a role in adipogenesis and/or lipogenesis and in macrophage biology. MNK1-KO/HFD mice show better glucose tolerance and insulin sensitivity, but gain weight and show similar adipose inflammation to WT animals. These data suggest MNK1 participates in mediating HFD-induced insulin resistance. Our findings reveal distinct roles for the MNKs in a novel area of disease biology, metabolic dysfunction, and suggests they are potential new targets for managing metabolic disease.

The consequences of over-consumption of food, in particular saturated fat, and other lifestyle factors pose a major and rapidly-increasing health problem worldwide. These consequences include obesity and associated conditions such as adipose tissue inflammation, insulin resistance and type 2 diabetes (T2D)^1^,^2^. Indeed, about 80% of cases of T2D can be attributed to obesity^3^. The number of people with T2D is increasing rapidly worldwide. Furthermore, obesity is associated with the ‘metabolic syndrome’, a major risk factor for cardiovascular disease, some types of cancer and depression^4^. These chronic disorders create major problems for affected individuals and a large, rapidly-increasing burden for health-care providers. It is crucial to gain a better understanding of the fundamental mechanisms through which high fat consumption leads to weight gain, inflammation and insulin resistance. This may pave the way for new approaches to managing obesity-linked inflammation and insulin resistance.

The murine MAP kinase-interacting kinases MNK1 and MNK2 are encoded by the *Mknk1* and *Mknk2* genes. The corresponding proteins (MNK1 and 2) interact with MAP kinases (e.g. ERK) and MNK1 binds p38 MAP kinase which is activated by inflammatory stimuli. MAP kinases phosphorylate the MNKs, resulting in their activation^5^. Their best-known substrate is translation initiation factor eIF4E, a key component of the protein synthetic machinery. Because eIF4E is encoded by a proto-oncogene^6^, most previous interest in the MNKs has focused on their potential roles in cancer^7^ Although closely related, MNK1 and MNK2 differ in a number of key respects; MNK1 is mainly cytoplasmic while MNK2 is also found in the nucleus^8^,^9^ and, while MNK1 is strongly activated following stimulation of the ERK or p38 MAP kinase pathway^10^,^11^,^12^, MNK2 shows high basal activity which is only slightly stimulated by these pathways. The expression of MNK1 and MNK2 in mouse tissues also differs, suggesting they play distinct roles^5^.

Here we have studied the roles of MNK1 and MNK2 in responses of C57BL/6J mice to consuming a high-fat diet (HFD), a widely-accepted model of HFD-induced metabolic disease^13^ using MNK- or MNK2-knockout (KO) animals. Disruption of one or both of the *Mknk1* and *Mknk2* genes has no reported adverse effects, even double-knockout mice being viable, fertile and without reported abnormalities^14^.

Our data reveal that HFD-fed MNK1-knockout (KO) and MNK2-KO mice are both protected against diet-induced glucose intolerance and insulin resistance compared to HFD-fed wild-type animals. Importantly, however, they are protected in distinct ways, indicating that MNK1 and MNK2 play distinct roles in metabolic disease. Our data demonstrate that these non-essential protein kinases are important in a major area of disease biology.

## Results

### MNK1 and 2 are expressed in mouse tissues involved in insulin-regulated metabolism

**Figure 1:**
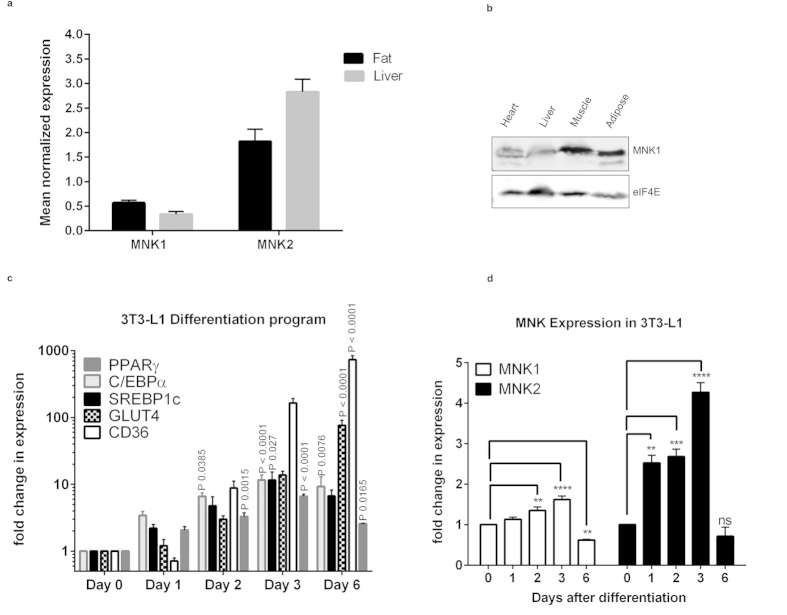
Analysis of *Mknk1* and *Mknk2* mRNA expression. (**a**) Relative expression of *Mknk1* (MNK1) and *Mknk2* (MNK2) was determined by qPCR in liver and gonadal adipose tissue (n = 8). Data are mean ± SEM. (**b**) Immunoblot analysis of MNK1 protein levels in liver, adipose tissue, heart and skeletal muscle; representative of 4 independent experiments. (**c**) mRNA expression of *PPARγ*, *C/EBPα*, *SREBP1c*, *GLUT4* and *CD36* in differentiating 3T3-L1 adipocytes relative to day 0. Data are mean ± SEM, one-way ANOVA with Tukey’s post-test (n = 3). (**d**) mRNA expression for *Mknk1* (MNK1) and *Mknk2* (MNK2) in differentiating 3T3-L1 adipocytes relative to day 0 (n = 3). Data are mean ± SEM (one-way ANOVA with Tukey’s post-test) **P < 0.01, ***P < 0.001, ****P < 0.0001.

Earlier studies showed the *MNK1* and *MNK2* mRNAs are expressed in liver, skeletal muscle and heart^5^, tissues involved in insulin-regulated metabolism. The *MNK1* and *MNK2* mRNAs are also expressed in adipose tissue (Fig. 1a). In contrast, *MNK2* is expressed only at low levels in some tissues, such as brain, where it makes only a small contribution to phosphorylation of eIF4E^15^. Immunoblot analysis revealed MNK1 protein expression in liver, skeletal and cardiac muscle and adipose tissue (Fig. 1b). As no suitable antibody is available for MNK2^16^, we cannot study its levels.

Given the high level of *MNK2* in adipose tissue we investigated the expression of *MNK2* during adipocyte differentiation, we used 3T3-L1 fibroblasts, a widely-employed model for this. Analysis of the expression of standard markers such as the transcription factors *PPARγ, C/EBPα* and *SREBP1c*, and the transporters *GLUT4* and *CD36*, confirmed the efficacy of the differentiation program (Fig. 1c). Levels of *MNK2* mRNA rose very quickly (at least as fast as the earliest of the other markers) (Fig. 1d), suggesting that MNK2 might play a role in adipocyte differentiation. The *MNK2* mRNA levels fell again by day 6. In contrast, *MNK1* mRNA levels changed only slightly.

The observation that MNK1 and MNK2 are expressed in tissues that play important roles in insulin-regulated metabolism prompted us to study the response of MNK1- or MNK2-KO animals to a HFD. So that we could define whether MNK1 and MNK2 play, perhaps different, roles in such responses, we used MNK1 and MNK2 single-knockout (KO) mice, rather than MNK1 + 2 double-KO mice.

### MNK2-KO mice are protected against HFD-induced fat gain and indices of insulin resistance

**Figure 2:**
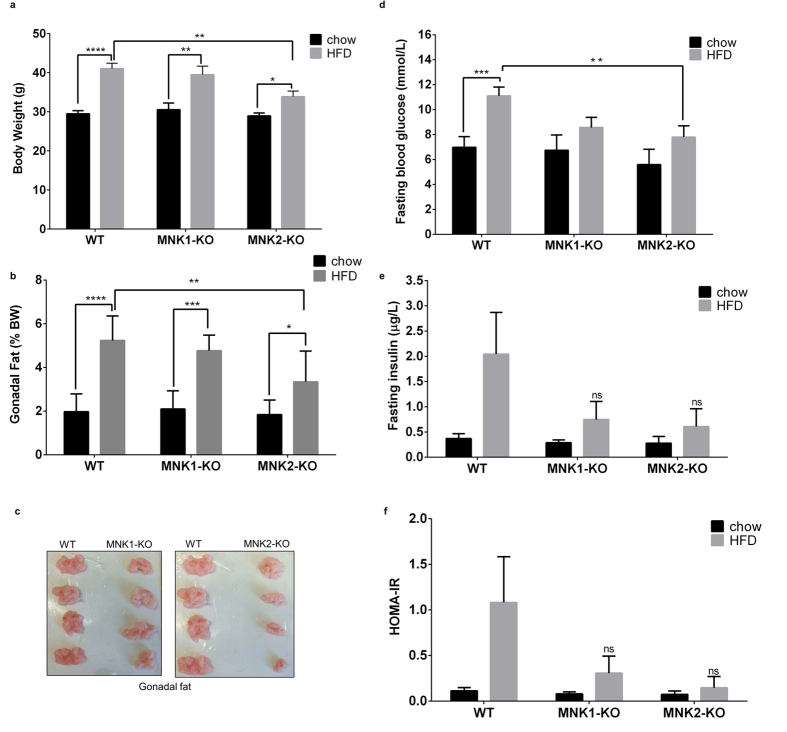
Responses of MNK1-KO and MNK2-KO mice to high fat feeding. (**a**) Bodyweight of WT, MNK1-KO and MNK2-KO mice after 20 weeks on either chow or a high fat diet (HFD) (45% kcal from fat) (n = 6–9). Data are mean ± SEM (two-way ANOVA followed by Tukey’s post test) *P < 0.05, **P < 0.01, ****P < 0.0001. (**b**) Gonadal adipose tissue weight expressed as a percentage of bodyweight (n = 6–9). Data are mean ± SEM (two-way ANOVA followed by Tukey’s post test) *P < 0.05, **P < 0.01, ***P < 0.001, ****P < 0.0001. (**c**) Representative images showing gonadal fat depots from WT, MNK1-KO and MNK2-KO mice after 20 weeks HFD. (**d**) Fasting blood glucose of WT, MNK1-KO and MNK2-KO mice after 20 weeks on either chow or a HFD (n = 6–9). Data are mean ± SEM (two-way ANOVA followed by Tukey’s post test) *P < 0.05, ***P < 0.001. (**e**) Fasting plasma insulin levels of WT, MNK1-KO and MNK2-KO mice after 20 weeks on either chow or a HFD (n = 3–5). Data are mean ± SEM. (**f**) Homeostasis model assessment of insulin resistance (HOMA-IR) as an index of insulin resistance.

As expected, feeding wild-type (WT) C57BL/6J mice an HFD led to increased bodyweight and gonadal fat (Fig. 2a–c). High fat-fed MNK1-KO mice showed similar increases in body and gonadal fat weight to WT mice on the HFD (Fig. 2a–c). In contrast, feeding MNK2-KO mice the same HFD caused smaller increases in body weight and fat. In WT mice, the HFD also caused marked increases in circulating levels of glucose and insulin (Fig. 2d,e, respectively). Such increases were markedly blunted in both MNK1-KO and MNK2-KO animals on the HFD, indicating attenuation of the adverse effects of the HFD. In chow-fed animals, basal glucose and insulin levels were similar to those in WT mice. We observed no differences in food intake of MNK-KO mice vs. wild-type controls.

The homeostatic model assessment of insulin resistance (HOMA-IR) is widely used as an index of insulin resistance^17^. As expected, the HOMA-IR was elevated in WT/HFD mice (Fig. 2f). However, in MNK2-KO/HFD mice, this value changed much less, indicating that they are largely protected from insulin resistance. Interestingly, although MNK1-KO mice show similar increases in weight and fat tissue to WT mice on the HFD, they also displayed a lower HOMA-IR than WT/HFD mice (Fig. 2d–f).

**Figure 3:**
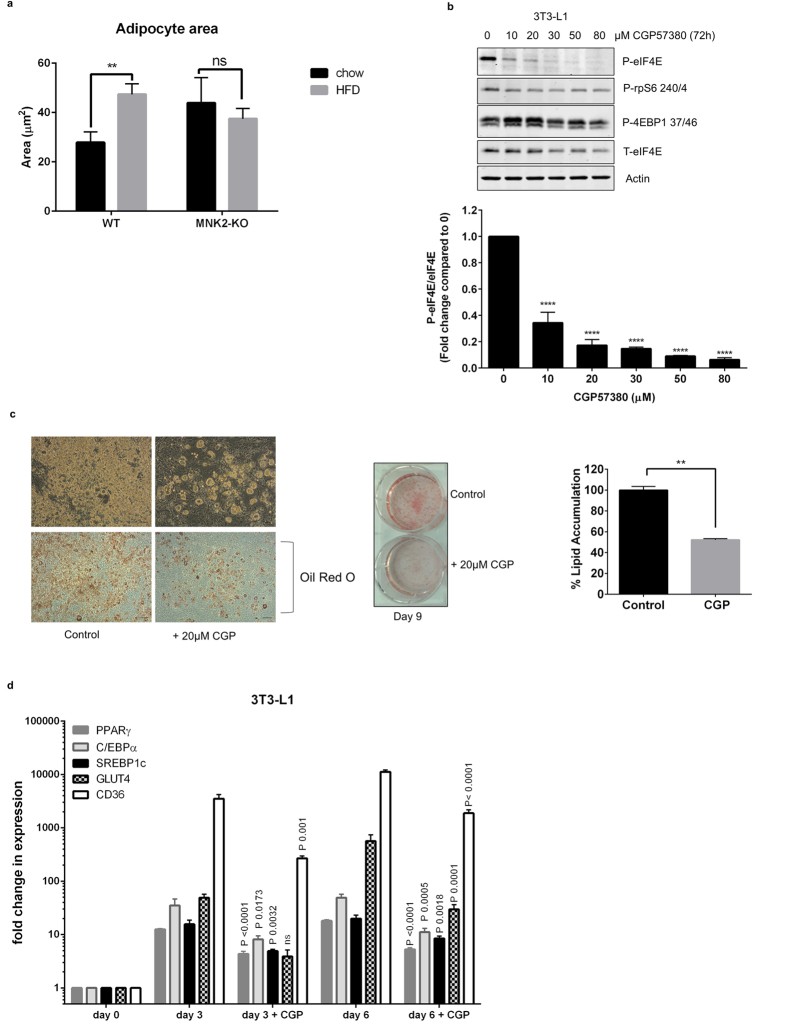
Effects of knocking out or inhibiting MNKs on adipocytes. (**a**) The average sizes of fat cells in WT and MNK2-KO mice fed chow or the HFD were analysed and quantified by image J software (WT chow n = 3, WT HFD n = 3, MNK2-KO chow n = 3, MNK2-KO HFD n = 3). Data are mean ± SEM (2-tailed, unpaired Student’s *t* test). **P < 0.01. (**b**) Right panel: undifferentiated 3T3-L1 cells were treated with the indicated concentrations of CGP57380 for 72 h. Lysates were analysed by immunoblot using the indicated antibodies, representative of 3 independent experiments. Below: Quantification of the data shown in the top panel. Data are mean ± SEM (one-way ANOVA with Tukey’s post-test) ****P < 0.0001. (**c**) 3T3-L1 cells were left to differentiate for 9 days in the absence or presence of 20 μM CGP57380 and cells were then stained with Oil Red O. Representative microscopic fields of view are shown, scale bars, 176 μm. Quantification of lipid incorporation by measurement of the intensity of Oil Red O staining (n = 3). Data are mean ± SEM relative to control (100%) (2-tailed, unpaired Student’s *t* test) **P < 0.01. (**d**) mRNA expression of *PPARγ*, *C/EBPα*, *SREBP1c*, *GLUT4* and *CD36* in 3T3-L1 adipocytes subjected to the differentiation program in the presence or absence 20 μM CGP57380 (n = 3). Data are mean ± SEM (one-way ANOVA with Tukey’s post-test).

In WT-HFD mice, adipocyte size increased substantially, as assessed by cell area (1.7 fold; Fig. 3a). Consistent with this, there was a corresponding decrease in the observed number of adipocytes seen in each field for HFD-fed WT compared to chow-fed mice. In contrast, adipocyte size in MNK2-KO/HFD mice did not increase when compared to chow-fed controls (Fig. 3a), suggesting there may be a defect in adipocyte lipid storage when these animals are fed the HFD. Interestingly, adipocyte size was larger in MNK2/chow animals than WT controls, suggesting that a possible deficit in adipogenesis in MNK2-KO mice, resulting in fewer adipocytes, each becoming bigger on the chow diet. The lack of increase in adipocyte size in MNK2-KO/HFD mice likely contributes to their blunted weight gain.

### MNKs are required for adipocyte differentiation

The blunted increase in fat tissue observed in the MNK2-KO/HFD mice compared to WT controls and the increased expression of *MNK2* during adipocytic differentiation (Fig. 1c) prompted us to examine whether MNKs play a role in adipocyte differentiation.

CGP57380 is a widely-used inhibitor of the activities of both MNK1 and MNK2^18^ and is superior to other available compounds^19^ such as cercosporamide which is very non-specific^16^. We therefore assessed the effect of CGP57380 on the differentiation of 3T3-L1 cells. We first conducted a dose-response study to determine the concentration of CGP57380 required to block MNK function, as assessed using phosphorylation of eIF4E as a read-out. (Since MNKs are the only kinases that phosphorylate eIF4E^15^, phosphorylation of eIF4E (P-eIF4E) provides a reliable read-out of MNK activity). This showed that 20 μM CGP57380 effectively blocked MNK activity in 3T3-L1 cells (Fig. 3b).

3T3-L1 cells were differentiated into adipocytes using a standard protocol; differentiation was monitored by assessing lipid content and evaluating adipocyte markers such as *PPARγ, C/EBPα, SREBP1c, GLUT4* and *CD36*.

At this concentration, CGP57380 inhibited the accumulation of lipid into 3T3-L1 cells subjected to the differentiation program (Fig. 3c). To study whether this reflected altered expression of genes involved in adipogenic differentiation, we examined the mRNA levels for *PPARγ*, *C/EBPα*, *SREBP1c*, *GLUT4* and *CD36*. CGP57380 substantially inhibited the induction of all the genes at both 3 and 6 days of the differentiation program (Fig. 3d). These data indicate that MNKs play a role in the differentiation of 3T3-L1 cells into adipocytes, and may therefore also play a role in adipocyte differentiation *in vivo*. This could potentially explain the data in Fig. 3a for adipocyte size and number.

### MNK2-KO/HFD mice do not show increased liver lipid accumulation

Given the attenuated gain in adipose tissue seen in MNK2-KO mice compared to WT animals on the HFD, it was important to assess whether the extra lipid load from the diet might be being diverted to the liver and contribute to fat accumulation there. MNK2-KO/HFD mice showed similar liver weights to WT mice on the HFD (Supplemental Fig. 1a,b). Total liver lipids were also similar for WT and MNK2-KO mice on the HFD (Supplemental Fig. 1c,d). These data suggest that the extra lipid from the HFD is not being re-directed to the liver in MNK2-KO mice.

### MNK-KO mice exhibit better glucose tolerance than WT mice on the HFD

**Figure 4:**
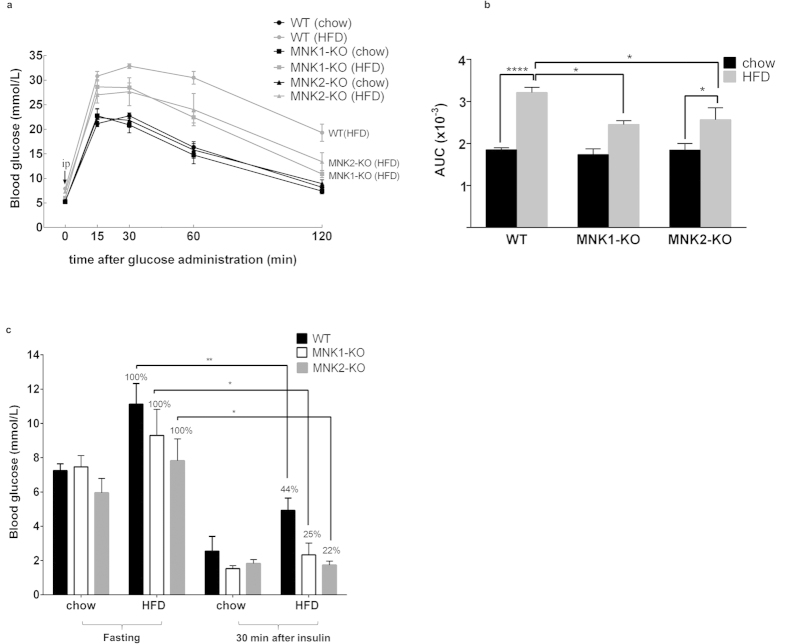
HFD-fed MNK1- or MNK2-KO mice show better glucose tolerance. (**a**) Glucose tolerance test. After 15 weeks on either chow or the HFD, mice were fasted overnight before receiving an intraperitoneal injection of 2 g/kg glucose, and blood samples were taken at the indicated times (n = 6–21). Data are mean ± SEM. (**b**) Area under the curve calculations (AUC) (n = 6–21). Data are mean ± SEM (two-way ANOVA with Tukey’s post-test). (**c**) Insulin action test. After 20 weeks on either chow or HFD, the mice were fasted overnight before receiving an intraperitoneal injection of 0.75 U/kg insulin, and blood samples were taken at 30 min (n = 3–4). Data are mean ± SEM (2-tailed, unpaired Student’s *t* test) *P < 0.05, **P < 0.01.

To assess directly the effect of the HFD on glucose handling in WT and MNK2-KO animals, we performed a glucose tolerance test (GTT). In the GTT, chow-fed MNK1- or MNK2-KO mice showed a similar response to WT animals indicating that the MNKs do not affect glucose handling under chow-fed conditions. However, MNK1- or MNK2-KO/HFD mice showed markedly lower blood glucose levels at all times after glucose administration relative to WT/HFD mice, which did show the expected marked impairment in glucose handling (Fig. 4a,b). Thus, knock-out of either MNK1 or MNK2 protects mice against HFD-induced glucose intolerance.

In order to assess insulin action, we assessed the ability of insulin decrease blood glucose levels in WT and MNK-KO mice on chow or HFD. Insulin decreased blood glucose levels significantly more effectively in MNK1- or MNK2-KO/HFD animals than in WT/HFD mice, indicating that insulin acts more efficiently in MNK-knockout/HFD mice (Fig. 4c), the percentage reduction in blood glucose caused by insulin in MNK1- or 2-KO/HFD mice being similar to that in chow-fed animals indicating insulin sensitivity is maintained in the KO mice.

Thus, MNK1-KO and MNK2-KO mice are each substantially protected against HFD-induced insulin resistance and glucose intolerance.

### MNK2-KO/HFD mice show better insulin signaling than WT/HFD animals

**Figure 5:**
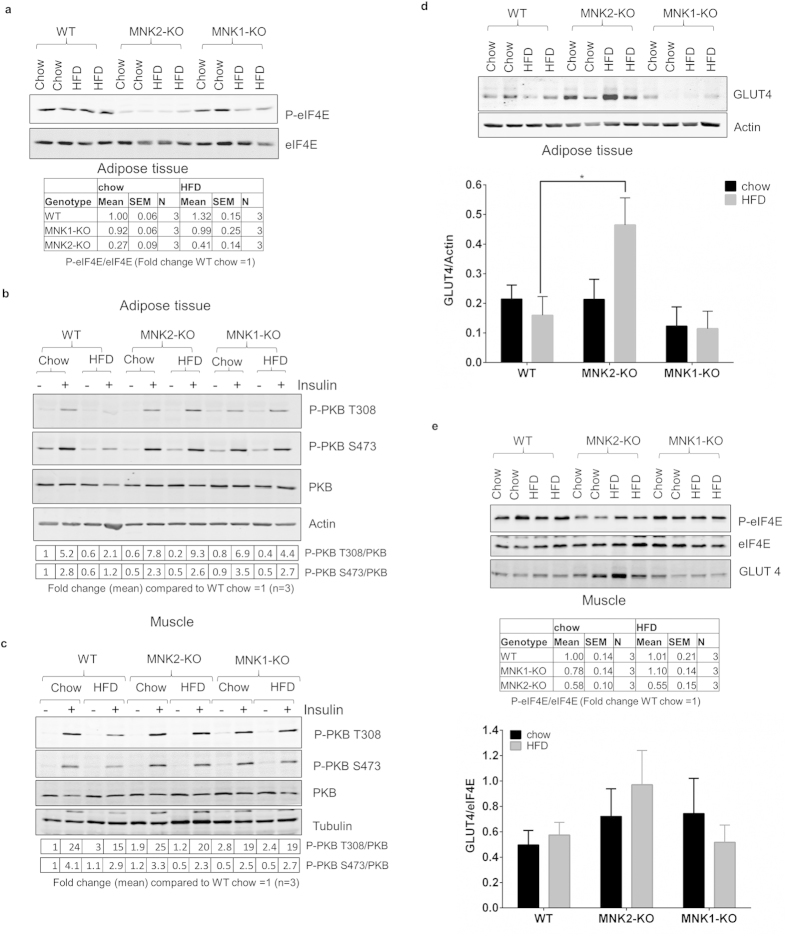
Analysis of signaling pathways in tissues from MNK-KO mice. (**a**) Total protein was isolated from gonadal adipose tissue from chow-fed and HFD-fed WT, MNK1-KO and MNK2-KO mice. Lysates were analysed by immunoblot using the indicated antibodies; representative of 3 independent samples. Below: quantification of the data shown in the top panel. Data are mean ± SEM, (n = 3). (**b**,**c**) Mice were fasted overnight before receiving an intraperitoneal injection of 0.75 U/kg insulin. Animals were sacrificed 30 min later, and tissues were collected for immunoblot analysis using the indicated antibodies. Representative immunoblot of WT, MNK1-KO and MNK2-KO adipose tissue (**b**) and skeletal muscle (**c**) are shown (n = 3). Below: quantification of the data shown in the top panel. Data are mean ± SEM, (n = 3). (**d**) Upper panel as in (**a**) lysates were analysed by immunoblot to assess GLUT4 levels. Lower panel: quantification of data from multiple experiments as in (**a**) expressed as GLUT4 normalized to actin. Data are mean ± SEM (2-tailed, unpaired Student’s *t* test comparing WT/HFD vs MNK2-KO/HFD or MNK1-KO/HFD) *P < 0.05. (**e**) as in (**a**,**e**) Upper panel: representative immunoblot of WT, MNK1-KO and MNK2-KO skeletal muscle is shown (n = 3). Below: quantification of the data shown in the top panel. Data are mean ± SEM, (n = 3). Lower panel: quantification of data from multiple experiments expressed as GLUT4 normalized to eIF4E.

To assess the relative contributions of MNK1/2 to overall MNK activity in adipose tissue, we examined phosphorylation of eIF4E, their common substrate^5^. The HFD caused a small increase in phosphorylated (P)-eIF4E (Fig. 5a) in WT mice but this did not reach statistical significance. MNK2-KO mice showed substantially lower P-eIF4E under both dietary conditions. MNK1-KO mice showed no change in P-eIF4E on the chow diet and the HFD caused only an insignificant decrease in P-eIF4E. This demonstrates that MNK2 is the major/most active MNK isoform in adipose tissue.

Insulin stimulates the uptake of glucose into tissues such as fat and especially muscle through the translocation of the glucose transporter GLUT4 to the plasma membrane^20^. This effect, like many metabolic effects of insulin, is mediated through protein kinase B (PKB, also termed Akt) which is phosphorylated and activated downstream of phosphatidylinositide 3-kinase (PI 3-kinase^21^). To assess insulin’s ability to activate this pathway, WT and MNK2-KO mice, fed either chow or the HFD, were treated with insulin and sacrificed 30 min later. Samples were taken of blood, adipose tissue and skeletal (gastrocnemius) muscle. Tissue samples were analysed by immunoblot for various parameters of insulin signaling.

In WT mice, as expected, insulin increased phosphorylation of PKB at Ser473 and Thr308, the main site involved in its activation, both in adipose tissue (Fig. 5b) and muscle (Fig. 5b,c). These effects were blunted in HFD-fed animals indicating the expected partial resistance to the effects of insulin, in line with a large body of earlier work. In MNK1- or MNK2-KO mice, insulin-induced PKB phosphorylation was similar on the chow diet, but higher on the HFD than in WT/HFD animals, showing that insulin resistance is attenuated in MNK-KO/HFD mice (Fig. 5b,c).

We observed an increase in total GLUT4 protein in adipose tissue of MNK2-KO/HFD mice compared to WT or chow-fed MNK2-KO animals (Fig. 5d). Thus, the improved glucose tolerance of MNK2 KO/HFD animals vs. WT/HFD mice may involve a combination of improved insulin sensitivity/signaling and higher GLUT4 protein levels. If anything, levels of *GLUT4* mRNA were actually slightly lower in adipose tissue of MNK2-KO animals (Supplemental Fig. 2), suggesting possible translational control of GLUT4 expression, whereby MNK2 would suppress synthesis of GLUT4 in line with the ability of MNKs to negatively regulate translation of certain other mRNAs^22^,^23^. GLUT4 protein levels were lower in adipose tissue of MNK1-KO animals (Fig. 5d).

In muscle, the major tissue involved in glucose disposal, P-eIF4E was decreased in MNK2-KO mice, but apparently maintained in MNK1-KO animals (Fig. 5e); this shows that MNK2 is the most active MNK isoform in this tissue. Insulin-induced PKB phosphorylation was blunted in HFD-fed WT mice, but not in MNK1- or MNK2-KO animals (Fig. 5e). GLUT4 protein levels tended to be higher in muscle of MNK2-KO mice, especially on the HFD, but the difference was not significant (Fig. 5e).

Thus, MNK1 and MNK2 both play roles in impairing insulin signaling in adipose tissue and muscle of HFD-fed mice.

### MNK2-KO mice are protected against HFD-induced adipose inflammation

**Figure 6:**
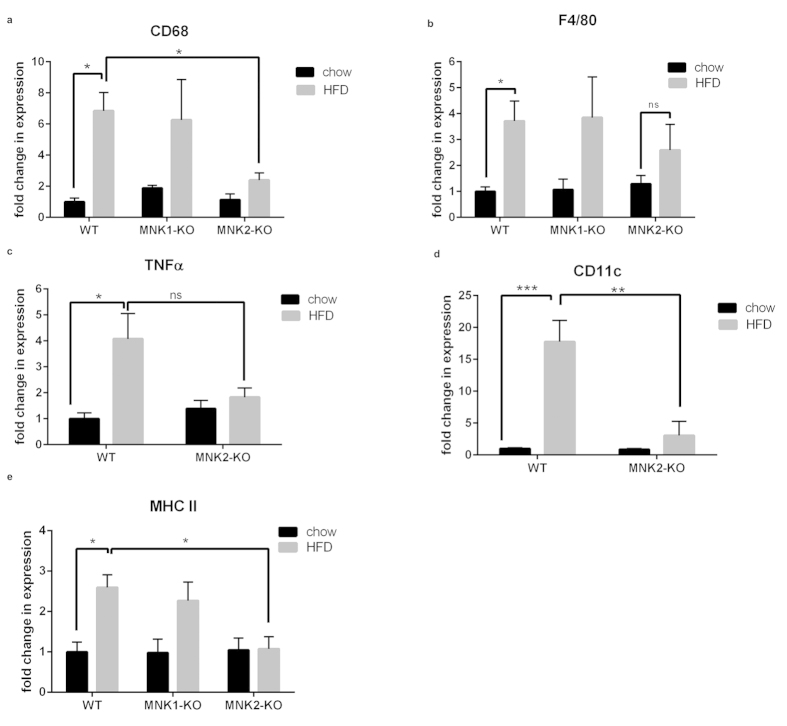
Analysis of macrophage markers in adipose tissue from WT and MNK-KO mice. (**a**,**b**) Total RNA was isolated from gonadal adipose tissue from chow-fed and HFD-fed WT, MNK1-KO and MNK2-KO mice. The relative expression of mRNA for general macrophage markers (*CD68* and *F4/80*) was measured by means of qPCR. Data are mean ± SEM relative to WT chow (2-tailed, unpaired Student’s *t* test) *P < 0.05, **P < 0.01. (**c**–**e**) The relative expression of mRNA for M1 polarized macrophages (*TNFα*, *CD11c* and *MHCII*) was measured by means of qPCR, (n = 3–4). Data are mean ± SEM relative to WT chow (two-way ANOVA followed by Tukey’s post test) *P < 0.05, **P < 0.01, ***P < 0.001.

A further major consequence of consuming a HFD is inflammation in adipose tissue, which becomes infiltrated with pro-inflammatory M1 macrophages^1^. WT/HFD and MNK1-KO/HFD mice both showed the expected increases, compared to chow-fed animals, in adipose tissue mRNA levels for macrophage markers such as *CD68* and *F4/80* (Fig. 6a,b). In marked contrast, MNK2-KO/HFD mice showed sharply blunted inflammation, with less or no increase in *CD68*, *F4/80* and *MHCII* (Fig. 6a,b,e).

In WT/HFD and MNK2-KO/HFD mice, we also examined the pro-inflammatory (M1 macrophage) markers *CD11c* and *TNFα* (Fig. 6c,d), the chemokine receptors *CCR2* and *CCR5* (important in macrophage trafficking; Supplemental Fig. 3a,b), and *MMP12* (matrix metalloproteinase 12) and *ADAM8*, which are involved in digesting extracellular matrix to allow cell migration (Supplemental Fig. 3c,d). As expected, the WT/HFD mice showed increases in all these markers. Such increases were significantly blunted in the MNK2-KO/HFD mice. Taken together, these data demonstrate that, on the HFD, the adipose tissue of MNK2-KO mice does not become heavily infiltrated with macrophages or show an increase in pro-inflammatory M1 macrophages. The lack of increase in *MHCII* in the MNK2-KO mice may be particularly relevant, as adipocyte MHCII plays a critical role in attracting immune cells to adipose tissue in HFD-fed animals^24^.

In summary, as expected, the HFD triggers extensive inflammation in adipose tissue of WT mice, while MNK2-KO mice are protected against the pro-inflammatory effects of the HFD. Interestingly, although MNK1-KO mice show partial protection against insulin resistance and glucose intolerance, they still exhibit similar adipose tissue inflammation to WT/HFD animals. This underscores the distinct roles that MNK1 and MNK2 play in responses to high fat feeding.

### Macrophage biology

The striking reduction in pro-inflammatory cytokine mRNAs in MNK2-KO/HFD VS. WT/HFD mice prompted us to assess whether MNK2-KO macrophages were intrinsically defective in producing cytokines such as TNFα and IL-6. Both MNK1 and MNK2 contribute to eIF4E phosphorylation in bone marrow-derived macrophages (BMDMs) (Supplemental Fig. 4a). Consistent with the MNKs’ regulatory characteristics^11^,^12^,^25^, the increase in eIF4E phosphorylation induced by lipopolysaccharide (LPS) in WT BMDMs was lost in MNK1-KO cells (Supplemental Fig. 4a). LPS increased the levels of the *TNF*α and *IL-6* mRNAs in WT and MNK2-KO BMDMs to similar extents (Supplemental Fig. 4b,c), indicating there is no intrinsic defect in the response of MNK2-KO BMDMs to LPS.

We also examined longer-term (24 h) effects of LPS and IFNγ to assess polarisation of BMDMs towards the pro-inflammatory M1 phenotype. BMDMs from WT and MNK2-KO mice responded similarly, indicating that MNK2 is not required for the production of these pro-inflammatory cytokines by BMDMs (Supplemental Fig. 4d,e).

**Figure 7:**
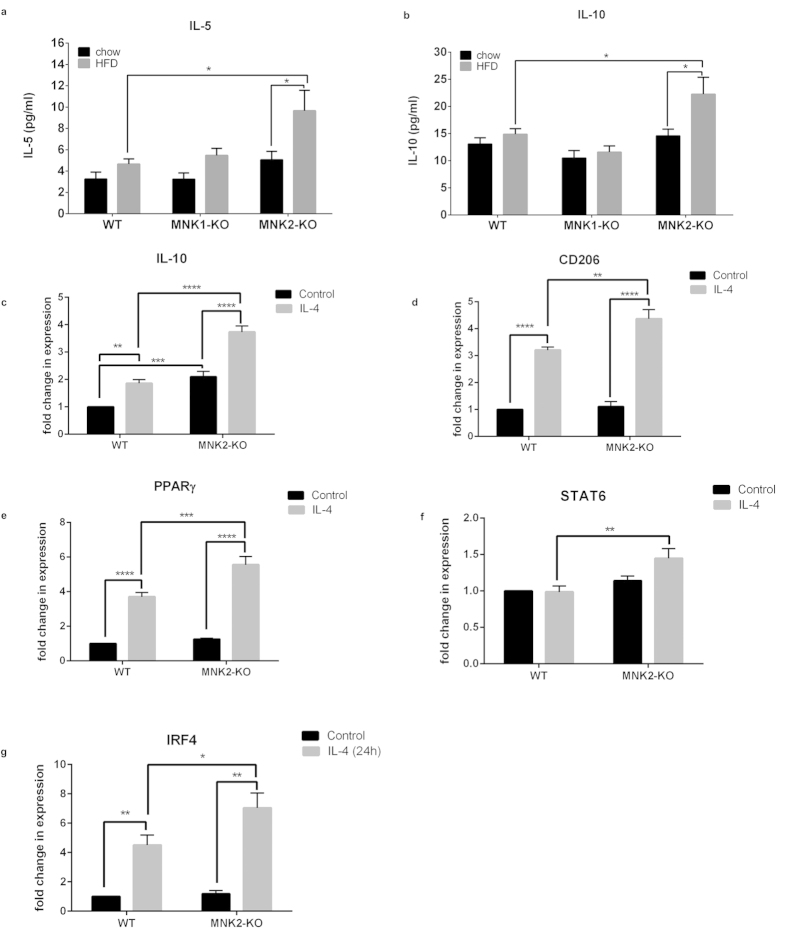
Analysis of marker genes in BMDMs from WT and MNK2-KO mice. (**a**,**b**) Plasma levels of IL-5 and IL-10 from chow-fed and HFD-fed WT, MNK1-KO and MNK2-KO mice were measured by ELISA (n = 6–9). Data are mean ± SEM (two-way ANOVA followed by Tukey’s post test) *P < 0.05. (**c**–**f**) BMDMs isolated from WT or MNK2-KO mice were cultured for 24 h in the presence or absence of IL-4 to polarize the BMDMs towards an M2 phenotype. The mRNA expression levels of the M2 markers *IL10*, *CD206*, *PPARγ*, *STAT6 and IRF4* were measured by qPCR (n = 6). Data are mean ± SEM relative to WT Control (two-way ANOVA followed by Tukey’s post test) *P < 0.05, **P < 0.01, ***P < 0.001, ****P < 0.0001.

We saw a significant increase in two anti-inflammatory markers, *IL-10* and *IL-5*, in the plasma of the MNK2-KO/HFD mice compared to WT/HFD mice (Fig. 7a,b). We therefore investigated the abilities of BMDMs from WT and MNK2-KO mice to polarize towards an M2 anti-inflammatory phenotype. Interestingly, we found that under control conditions, MNK2-KO BMDMs had elevated levels of *IL-10* and *PPARγ* compared to WT BMDMs. All M2 markers examined were increased more in these cells than in WT-BMDMs after 24 h stimulation with IL-4 (Fig. 7c–g). The data suggest that MNK2-KO macrophages have a higher tendency towards an anti-inflammatory phenotype.

### High-fat feeding induces Nrf2 expression in MNK2-KO but not wild-type mice

**Figure 8:**
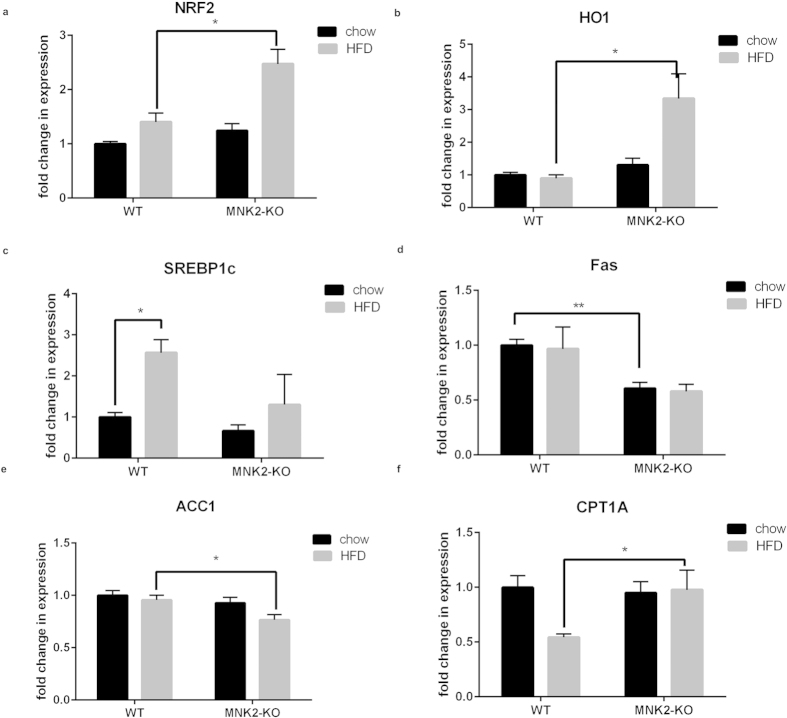
Analysis of expression of selected genes in liver of WT and MNK2-KO mice. (**a**,**b**) Total RNA was isolated from liver tissue from chow-fed and HFD-fed WT and MNK2-KO mice. The relative expression of *NRF2* and its downstream target heme oxygenase 1 (*HO1*) was measured by means of qPCR (n = 4). Data are mean ± SEM (2-tailed, unpaired Student’s *t* test comparing WT/HFD vs MNK2-KO/HFD) *P < 0.05. (**c**–**f**) The relative expression of genes involved in *de novo* lipogenesis (*SREBP1c, FAS, ACC1*) and β-oxidation (*CPT1A*) in the liver from chow-fed and HFD-fed WT and MNK2-KO mice were measured by qPCR (n = 4). Data are mean ± SEM relative to WT chow (2-tailed, unpaired Student’s *t* test) *P < 0.05, **P < 0.01.

Nuclear factor erythroid 2-related factor 2 (Nrf2) is important in protection against oxidative stress^26^, which can arise from extensive fatty acid oxidation^27^. Nrf2 is reported to protect against development of the metabolic syndrome (reviewed^26^) and steatosis^28^. Given that MNK2-KO mice show protection against indices of metabolic syndrome, we analysed the expression of *Nrf2* in livers of WT and MNK2-KO mice. Levels were similar in mice fed a chow diet. *Nrf2* mRNA levels increased markedly in MNK2-KO mice fed a HFD, but not in the corresponding control animals (Fig. 8a). To confirm increased Nrf2 function, we tested expression of haem oxygenase-1 (*HO-1*; an Nrf2 target gene), which showed a similar pattern to *Nrf2* (Fig. 8b). Nrf2 can be transcriptionally controlled by PPARα^29^; however, we saw no differences in PPARα levels in liver of MNK2-KO or WT mice on the HFD (Supplemental Fig. 5c).

### Hepatic gene expression in MNK2-KO mice

Fatty liver disease is strongly associated with high dietary intake of fat and/or energy^30^. SREBP1c is a transcription factor important for hepatic lipogenesis; its levels were markedly increased in livers of WT/HFD but not MNK2-KO/HFD mice (Fig. 8c). In contrast, levels of *LXR* (an upstream regulator of SREBP1c) were unaltered (Supplemental Fig. 5a). Fatty acid synthase (*FAS*) and acetyl-CoA carboxylase 1 (*ACC1*) are key lipogenic enzymes. The levels of *FAS* mRNA, an SREBP1c target gene, were lower in liver of MNK2-KO mice under both conditions, and *ACC1* decreased on the HFD in MNK2-KO but not WT mice (Fig. 8d,e, respectively). ATP-citrate lyase (*ACYL*) catalyzes the formation of acetyl-coenzyme A (acetyl-CoA, precursor for fatty acid synthesis). Expression of *ACYL* is also controlled by the SREBP1c. *ACYL* mRNA levels were lower in livers of MNK2-KO, but not WT mice, on the HFD (Supplemental Fig. 5b). These data suggest that MNK2-KO mice are relatively protected against increased hepatic lipogenesis on an HFD.

We also examined the levels of mRNAs for proteins involved in hepatic lipid oxidation. Carnitine palmitoyltransferase 1 A (CPT-1 A) catalyses a key step in transport of fatty acids into mitochondria for β-oxidation. Whereas *CPT1A* mRNA levels fell in WT mice on the HFD^31^ (thus tending to favour fatty acid storage over oxidation), they were unchanged in MNK2-KO animals fed the HFD (Fig. 8f).

Given the data suggesting a preference for fatty acid breakdown and oxidation rather than storage in MNK2-HFD mice, we looked at expression in adipose tissue levels of hormone-sensitive lipase mRNA (*HSL*), a key lipolytic enzyme. They fell in WT mice on the HFD, but not in MNK2-KO/HFD animals (Supplemental Fig. 6a), consistent with maintained rates of lipid breakdown in them.

As expected, treatment of differentiated 3T3-L1 cells with the β-adrenergic agonist isoproterenol increased glycerol production, indicating enhanced lipolysis (Supplemental Fig. 6b). Less glycerol was released from cells differentiated in the presence of CGP57380 under basal or stimulated conditions, consistent with decreased storage of triglyceride in the CGP57380-treated cells (cf. data in Fig. 3d). Short-term CGP57380 treatment did not affect glycerol output in 3T3-L1 cells that had been differentiated under control conditions (Supplemental Fig. 6b) showing that MNK inhibiting does not block lipolysis *in vitro*.

## Discussion

**Figure 9:**
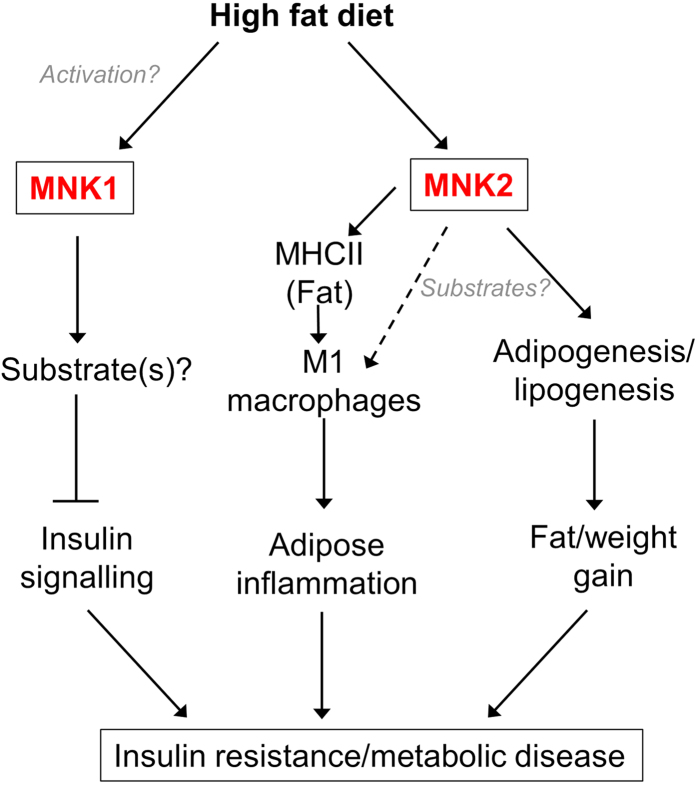
Model for the role of MNK1 and MNK2 in mediating the adverse effects of a high fat diet. Please see the Discussion for further information.

The data presented here reveal that knocking out expression of the MNK protein kinases results in a substantial blunting of the several major adverse effects of a HFD, which include weight gain, inflammation and insulin resistance (Fig. 9). This is the first report linking the MNKs to metabolic disease. Following HFD-feeding, MNK1- or MNK2-KO mice show substantially better glucose tolerance and insulin responsiveness than the corresponding WT mice. These data imply that MNK1 and MNK2 each play key roles in in the deleterious consequences of HFD feeding.

Intriguingly, there are striking differences between the phenotypes of the HFD-fed MNK1- and MNK2-KO mice; while HFD-fed MNK2-KO show reduced weight gain and greatly diminished adipose tissue inflammation, MNK1-KO mice show similar increases in fat mass and inflammatory markers to WT animals on the HFD, but are nonetheless protected to a similar extent against HFD-induced insulin resistance and glucose intolerance. Thus, MNKs play important but quite distinct roles in the responses of mice to a HFD (see Fig. 9 for a summary).

These differences could reflect the distinct regulatory properties and/or tissue distribution of MNK1 and MNK2. For example, MNK2 is the major MNK isoform in adipose tissue and, unlike MNK1, has high basal activity which is only slightly enhanced by these upstream signaling pathways^10^. Our data indicate that MNKs are involved in the differentiation of precursor cells into adipocytes; since MNK2 appears to be strongly induced in these cells, it is likely to play the major role in this process (see Fig. 9). Defects either in the differentiation of adipocytes or in lipid metabolism could underlie the protection displayed by MNK2-KO animals against HFD-induced weight/fat gain.

We also observed differences in the expression of genes for lipogenic or lipolytic enzymes between HFD-fed WT and MNK2-KO mice which point to a shift towards lipolysis rather than lipogenesis in the liver of MNK2-KO/HFD mice relative to WT/HFD mice. This is consistent with the absence of an increase in average adipocyte size in MNK2-KO mice on the HFD.

In the case of MNK2-KO mice, we observed a substantial attenuation of the increases in macrophage markers in the adipose tissue of HFD-fed mice and a very strong reduction in the levels of pro-inflammatory markers (associated with M1 macrophages). It may be particularly important that, whereas WT mice on the HFD show a marked increase in *MHCII* in adipose tissue, MNK2-KO/HFD mice do not, since recent data suggest that adipocyte MHCII plays a key role in recruiting immune cells leading to adipose inflammation in HFD-fed animals^24^. Indeed, MHCII-KO mice show strong protection against HFD-induced inflammation and insulin resistance. The absence of an increase in the expression of MHCII therefore likely contributes to the blunting of the inflammatory response in MNK2-KO/HFD animals^24^.

In contrast, analysis of macrophage markers in adipose tissue of MNK1-KO/HFD mice revealed similar levels to WT/HFD mice. Thus, while MNK2 plays an important role in HFD-induced adipose inflammation, MNK1 does not. Interestingly, MNK2-KO mice showed higher levels of anti-inflammatory cytokines (IL-5 and IL-10). BMDMs from MNK2-KO animals also showed elevated basal levels of M2 markers and an increased tendency towards M2 polarisation in response to IL-4, suggesting that MNK2-KO cells are skewed towards the M2 phenotype.

Knockout of MNK2 protects mice against HFD-induced insulin-resistance. This is clear from our data showing lower circulating blood insulin and glucose levels in MNK2-KO/HFD animals vs. WT/HFD mice, their improved glucose tolerance and their better response to administration of insulin (in terms of lowered blood glucose levels). This protective effect of the MNK2-KO signaling may well be linked to the attenuation of inflammation (since pro-inflammatory cytokines can impair insulin signaling, see^32^). An HFD can cause insulin resistance via activation of c-Jun amino-terminal kinase (JNK)^33^,^34^. However, the MNKs are not activated by JNK^5^ ruling out a role for that pathway in the phenotype of MNK-KO/HFD mice.

The increase in GLUT4 protein levels observed in MNK2-KO mice, and a consequent uptake of glucose into adipose tissue, could lead to an increase in formation of triacylglycerols (TAG). However, we did not observe increased fat deposition in MNK2-KO/HFD mice. This may reflect, e.g. the maintained expression of lipolytic genes such as *HSL*.

MNK2-KO mice also showed increased expression of the transcription factor Nrf2 in liver, perhaps reflecting increased lipid oxidation. Nrf2 can protect against HFD-induced metabolic syndrome (reviewed^26^), also ameliorating the response of these animals to the HFD. Further work is clearly required to define the roles of MNK2 in regulating lipid metabolism and adipocyte differentiation and the precise mechanisms involved in this.

In the case of MNK1-KO animals, the HFD-induced weight/fat gain and inflammatory responses were similar to those in WT animals. The lack of effect of the MNK1-KO in adipose tissue gain may reflects the fact that MNK2, not MNK1, is the major isoform in this tissue. The fact that MNK1-KO mice are nonetheless protected against glucose intolerance and insulin-insensitivity suggests that MNK1 may regulate peripheral insulin sensitivity. Indeed, MNK1 is activated by pro-inflammatory cytokines such as those which increase in metabolic disease (e.g.^12^). One well-studied mechanism by which insulin signaling can be controlled is through the phosphorylation of insulin receptor substrate (IRS) 1^35^. However, in preliminary data we did not observe direct phosphorylation by MNK1 of recombinant fragments of IRS1 *in vitro* (data not shown).

Our data show that MNK1 and MNK2 each contribute to the adverse effects of HFD feeding, and that they do so in distinct ways (Fig. 9). These kinases share a well-known substrate, eIF4E, prompting the question: can the observed phenotypes can be explained entirely through the loss of phosphorylation of this substrate? eIF4E phosphorylation is decreased in fat from MNK2-KO mice, indicating that MNK2 is a major MNK isoform in that tissue. In this tissue, GLUT4 protein levels in MNK2-KO mice are elevated compared to the WT animals whereas the mRNA levels are actually lower. This suggests that MNK2 may regulate translation of the *GLUT4* mRNA, consistent with MNK2 normally suppressing translation of this message, as is the case for some other mRNAs^22^,^23^. The increased levels of GLUT4 protein likely contribute to the improved glucose tolerance in MNK2-KO/HFD mice.

Muscle is the main tissue involved in peripheral glucose uptake. eIF4E phosphorylation is unaffected in muscle from MNK1-KO mice indicating that MNK1 makes a minor contribution to overall eIF4E phosphorylation in this tissue and implying that effects of loss of MNK1 in muscle are unlikely to be due to altered eIF4E phosphorylation. The MNKs have additional *in vitro* substrates (reviewed in^6^) and their substrate specificities appear to differ^36^. However, none of those substrates has any obvious connection to insulin signaling. We are therefore currently screening for novel MNK substrates.

Since our data reveal that loss of MNK1 or MNK2 protects against HFD-induced insulin resistance and glucose intolerance in distinct ways, we predict that MNK1/2 double-knockout animals will show a stronger protection against these adverse effects of the HFD than mice knocked out for either MNK alone.

Given that MNK1/2 double-KO mice show no overt phenotype under controlled vivarium conditions^14^, the MNKs are attractive therapeutic targets. Importantly, these data imply that interfering with the function of MNK1 and MNK2 using, for example, a small molecule inhibitor, may help prevent or diminish the effects of HFD feeding, which lead to a range of highly prevalent chronic diseases.

Ultimately, when compounds suitable for inhibiting MNK activity *in vivo* become available, it will be important to explore the value of inhibiting the MNKs in tackling the effects of an HFD in WT mice. Indeed, inhibitors of the MNKs may be valuable new therapeutic agents for metabolic disease.

## Methods

### Materials and chemicals

All cell culture solutions and supplements were purchased from Life Technologies. Reagents for SDS-PAGE were purchased from Bio-Rad. For the adipogenesis experiment, insulin, dexamethasone, IBMX and rosiglitazone were obtained from Sigma. For macrophage polarization, lipopolysaccharide (LPS; L2630-lipopolysaccharides from *Escherichia coli* 0111:B4) was purchased from Sigma Aldrich, interleukin (IL)-4 and interferon (IFN) γ were purchased from Peprotech. The Mnk inhibitor CGP57380 was obtained from Abcam.

### Animal use and diet

All animal procedures were conducted at the University of Southampton in accordance with the regulations of the United Kingdom Animals (Scientific Procedures) Act 1986 and were conducted under Home Office Project Licence number 30-2968. The study received institutional approval from the University of Southampton Biomedical Research Facility Research Ethics Committee. The previously-generated general MNK1-KO and MNK2-KO mice were created on a C57BL/6J background and kindly provided by Dr Rikiro Fukunaga (Osaka University, Japan^14^).

Wild-type (WT), MNK1-KO or MNK2-KO mice were kept under a 12-h light/dark cycle (lights on at 07:00 h) and at a constant temperature of 22 ± 2 °C with food and water available ad libitum. At four weeks, they were allocated to either a high fat diet (HFD) diet (45% kcal fat, 20% kcal protein, 35% kcal carbohydrate; Special Dietary Services) or a standard chow diet (7% kcal fat, 18% kcal protein, 75% kcal carbohydrate; Special Dietary Services) for 20 weeks.

### Metabolic studies

After 15 weeks on either the chow or HFD a glucose tolerance test (GTT) was performed after an overnight fast. Fasting glucose concentrations were measured from whole blood obtained from the tail vein before the mice were intraperitoneally (i.p.) injected with D-glucose (2 g/kg body weight; Baxter Healthcare Ltd), and blood glucose concentrations were measured in samples from the tail vein at 15, 30, 60 and 120 min post i.p. injection using an Aviva Accu-Chek glucometer (Roche Diagnostics).

To test for insulin resistance, a separate cohort of mice was fasted overnight and fasting glucose concentration in whole blood obtained from the tail was measured before i.p. insulin injections (0.75 U/kg mouse body weight; Actrapid, Novo Nordisk). Blood glucose concentrations were then measured from the tail vein at 15 and 30 min after i.p. injection. Mice were immediately sacrificed after taking the second (30-minute) glucose reading and tissues were frozen immediately for analysis by Western blotting to measure downstream insulin signaling.

Measurements of plasma insulin, IL-5 and IL-10 were performed by ELISA at the Core Biochemical Assay Laboratory (CBAL) in Cambridge, UK.

### Western blotting

Tissues were harvested in RIPA lysis buffer containing 50 mM Tris-HCl, pH 7.4, 150 mM NaCl, 1% Triton X-100, 0.1% sodium deoxycholate, 0.1% sodium dodecyl sulfate, 1 mM ethylenediaminetetraaacteic acid (EDTA), 50 mM β-glycerophosphate, 0.5 mM NaVO_3_, 0.1% 2-mercaptoethanol and protease inhibitors (Roche). After lysis, insoluble material was removed by centrifugation at 12,000 *g* for 10 min at 4 °C. Protein content was determined by the Bradford protein assay (Bio-Rad). Immunoblotting was performed as described^37^. Blots were visualized using a LI-COR Odyssey Quantitative Imaging System. Primary antibodies were from Cell Signaling Technology, except: P-eIF4E (Merck Millipore). Secondary antibodies were obtained from Fisher Scientific and used at 1:20,000 dilution.

### Gene expression analysis

Total RNA was isolated from tissues (that had been frozen at the time of sampling) using Trizol reagent (Sigma Aldrich) according to the manufacturer’s instructions. RNA concentrations were determined by absorbance at 260 nm, while quality and integrity were evaluated via 260/230 and 260/280 ratios using using a NanoDrop spectrophotometer (Thermo Fisher Scientific). RT-real time PCR amplification was carried out using the ImProm-II Reverse Transcription System (A3800 Promega) with random primers following the manufacturer’s protocol. Subsequently, real-time quantitative (q) PCR was performed (see Supplemental Table 1 for primer sequences). The comparative *C*_T_ method was used to measure amplification of target mRNA levels compared with β_2_ microglobulin (B2M) mRNA. In Fig. 1a, the relative amount of each transcript was determined using the equation 2^−dCt^, where dCt = (Ct target gene–Ct ref gene).

### Isolation of BMDMs

Bone marrow-derived macrophage (BMDMs) were generated as described previously^38^ from adult WT or MNK2-KO mice. Briefly, bilateral femurs and tibias of mice were flushed using 26-gauge needles into sterile Hank’s Balanced Salt Solution (HBSS) without Ca^2+^ and Mg^2+^ (Life Technologies). Cell clusters were disrupted by pipetting and passing the suspension through a 40 μm cell strainer. The resulting cell suspension was centrifuged (10 min, 400 × g) and the cell pellet was resuspended in complete macrophage medium (CMM) containing 30% L929 cell conditioned medium (L929 cells secrete macrophage colony-stimulating factor (M-CSF) required for the promotion of bone marrow cell differentiation into macrophages), 20% fetal bovine serum (FBS), 50% DMEM (high glucose Dulbecco’s Modified Eagle Medium; DMEM, Life Technologies) and with 1% (w/v) penicillin/streptomycin (Life Technologies).

### Macrophage polarization

To promote polarization into M1 or M2 macrophages, BMDMs were treated with LPS (100 ng/ml) plus interferon (IFN)-γ (20 ng/ml; Peprotech) or IL-4 (20 ng/ml; Peprotech), respectively, for 24 h.

### Differentiation of 3T3-L1 cells and Oil red O staining

For induction of 3T3-L1 (fibroblast) cell differentiation, preadipocytes were grown to 2 days post-confluence in DMEM supplemented with 10% FBS (day 0) and the medium was changed to DMEM supplemented with 10% FBS, insulin (167 nM), dexamethasone (0.5 μM), isobutylmethylxanthine (IBMX) (0.5 mM) and rosiglitazone (2 μM). After 48 h, the medium was replaced with medium containing DMEM supplemented with 10% (v/v) FBS and 167 nM insulin. On day 4, after inducing differentiation, and thereafter, the cells were cultured in DMEM with 10% FBS. The maintenance medium was changed every 48 h until the cells were utilized for experimentation (9 days from the initiation of the differentiation program).

Oil Red O staining and measurement of intracellular triglyceride levels were performed as described by others^39^. For quantification, cells were washed extensively with water to remove unbound dye, and 1 ml isopropanol was added to the stained 6-well culture plate. After 5 min, the absorbance of the oil red extract was measured at 510 nm.

### Histology

Adipose tissue sections were fixed in 10% neutral buffered formalin for 6 h and dehydrated as standard before embedding in paraffin wax. Sections (4 μm) were cut and mounted on positively-charged glass slides and hematoxylin and eosin (H&E) staining was performed as standard. Slides were scanned using the Pannoramic 250 Flash II scanner (3DHISTECH, Budapest, Hungary). Images were analysed using Image J with the adipocyte tool macro. Adipocytes were then counted, and the absolute pixel area of each object was calculated and converted to μm^2^.

### Statistics

Analysis was performed by 2-tailed, unpaired Student’s *t* test, one-way or two-way ANOVA as indicated in the figure legends. A *P* value less than 0.05 was considered significant. Statistical tests were performed using the statistical program GraphPad Prism (ver. 6; GraphPad Software Inc.).

### Lipolysis assay

3T3-L1 cells were differentiated as described in the methods section for 9 days. The lipolysis assay was performed according to the manufacturer’s instructions (Abcam Lipolysis assay kit, ab185433). Briefly, after differentiation cells were washed two times with lipolysis assay buffer. Lipolysis was stimulated using 100 nM isoproterenol for 3 h. The amount of glycerol released was measured using colorimetric intensity.

### Triglyceride measurement

The amount of tissue-released triglyceride (triacylglycerol; TAG) was measured using a kit from Abcam (Triglyceride Quantification Kit, ab65336).

### Liver histology

Fresh frozen sections (5 μm thick) were used to detect lipid accumulation by staining with Oil Red O (Sigma). Cryosections were fixed in 60% isopropanol for 10 min and stained with 0.3% Oil Red O in 60% isopropanol for 30 min and subsequently washed with 60% isopropanol. Nuclei were stained with hematoxylin for 2 min and then rinsed with tap water. Slides were scanned using the Pannoramic 250 Flash II scanner (3DHISTECH, Budapest, Hungary).

## Additional Information

**How to cite this article**: Moore, C. E. J. *et al.* MNK1 and MNK2 mediate adverse effects of high-fat feeding in distinct ways. *Sci. Rep.* **6**, 23476; doi: 10.1038/srep23476 (2016).

## Supplementary Material

Supplementary Information
