# Supplementary material for: MNK1 and MNK2 mediate adverse effects of high-fat feeding in distinct ways
Source: Sci Rep. 2016 Apr 18;6:23476. doi: 10.1038/srep23476 (PMC4834573; doi:10.1038/srep23476)
Supplement: Supplementary Information [file srep23476-s1.pdf]

Supplementary Information

MNK1 and MNK2 mediate adverse effects of high-fat feeding in distinct ways

[1,2]Moore, C.E.J., [1]Pickford, J., [3]Cagampang, F.R., [1]Stead, R.L., [1,2]Tian, S., [2]Zhao, X., [4]Tang, X., [5,6]Byrne, C.D., [1,2,7]*Proud, C.G.

[1] Centre for Biological Sciences, University of Southampton, Southampton, SO17 1BJ, United Kingdom

[2] Nutrition and Metabolism, South Australian Health & Medical Research Institute, North Terrace, Adelaide, SA5000, Australia

[3] Institute of Developmental Sciences, University of Southampton, Faculty of Medicine, Southampton SO16 6YD, United Kingdom

[4] Department of Biochemistry & Genetics, Zhejiang University School of Medicine, Hangzhou, Zhejiang 310058 China

[5] Nutrition and Metabolism, University of Southampton, Faculty of Medicine, Southampton SO16 6YD, United Kingdom

[6] Southampton National Institute for Health Research, Biomedical Research Centre, University Hospital, Southampton, UK

[7] School of Biological Sciences, University of Adelaide

* present address, for correspondence: Nutrition & Metabolism, South Australian Health & Medical Research Institute, North Terrace, Adelaide, SA5000, Australia;

email: christopher.proud@sahmri.com

Table S1
Sequence of primers used in RT-qPCR

Gene	Forward	Reverse
**Mknk 1 (MNK1)**	GATTCCTCTGAGACTCCAAGTTAA	ACGCTTCTTCTTCCTCCTCTT
**Mknk 2 (MNK2)**	CCAGTGCCAGGGACATAGG	GCCACGCATCTTCTCAAACA
**ACC1**	GCAGTCTCCCAACTCCTACG	CCACCATCACTCAGCCGAT
***ATP citrate lyase* (*ACLY*)**	CAGACCTATGACTATGCCAAGAC	CAATGCTGCCTCCAATGATGA
GLUT4	AAAAGTGCCTGAAACCAGAG	TCACCTCCTGCTCTAAAAGG
**CPT1a**	AAACCTATTCGTCTTCTGGGATC	ATGTGCCTGCTGTCCTTGA
**LXRα**	ATCGCCTTGCTGAAGACCTCTG	GATGGGGTTGATGAACTCCACC
**CCR2**	ATTCTCCACACCCTGTTTCG	GATTCCTGGAAGGTGGTCAA
**CCR5**	CGAAAACACATGGTCAAACG	GTTCTCCTGTGGATCGGGTA
**SREBP1c**	GGAGCCATGGATTGCACATT	CCTGTCTCACCCCCAGCATA
**HSL**	CTC CAC ATG CCCCTC TAC AC	CAGAGCGCAAGCCACAAG
**NRF2**	TCCGCCAGCTACTCCAGGTTGG	TGGGCCCTGATGAGGGGCAGTG
**C/EBPα**	GAACAGCAACGAGTACCGGGTA	GCCATGGCCTTGACCAAGGAG
PPARγ	TCCGTGATGGAAGACCACTCGCAT	CAGCAACCATTGGGTCAGCTCTTG
**Heme oxygenase 1 (HO-1)**	CCTCACTGGCAGGAAATCATC	CCTCGTGGAGACGCTTTACATA
CD36	TCATGCCAGTCGGAGACATGCTTA	AACTGTCTGTACACAGTGGTGCCT
IL-6	TCCATCCAGTTGCCTTCTTG	GGTCTGTTGGGAGTGGTATC
IL-10	GCTCTTACTGACTGGCATGAG	CGCAGCTCTAGGAGCATGTG
**CD11c**	CTGGATAGCCTTTCTTCTGCTG	GCACACTGTGTCCGAACTC
***TNF*-α**	TCTCAGCCTCTTCTCATTCCTGCT	AGAACTGATGAGAGGGAGGCCATT
**MMP12**	TTGGATTATTGGAATGCTGC	GCACATTTTGATGAGGCAGA
**CD68**	TTCTGCTGTGGAAATGCAAG	AGAGGGGCTGGTAGGTTGAT
**CD206**	CAGGTGTGGGCTCAGGTAGT	TGTGGTGAGCTGAAAGGTGA
**STAT6**	CTGGGGTGGTTTCCTCTTG	TGCCCGGTCTCACCTAACTA
**F4/80**	CTTTGGCTATGGGCTTCCAGTC	GCAAGGAGGACAGAGTTTATCGTG
**MHCII**	GACGCTCAACTTGTCCCAAAAC	GCAGCCGTGAACTTGTTGAAC
**ADAM8**	AGGATATTCAGCAGGTGTAGCAA	TGCTAAAGGTATAGCAGGAGTCG
**B2M**	CTGCTACGTAACACAGTTCCACCC	CATGATGCTTGATCACATGTCTCG

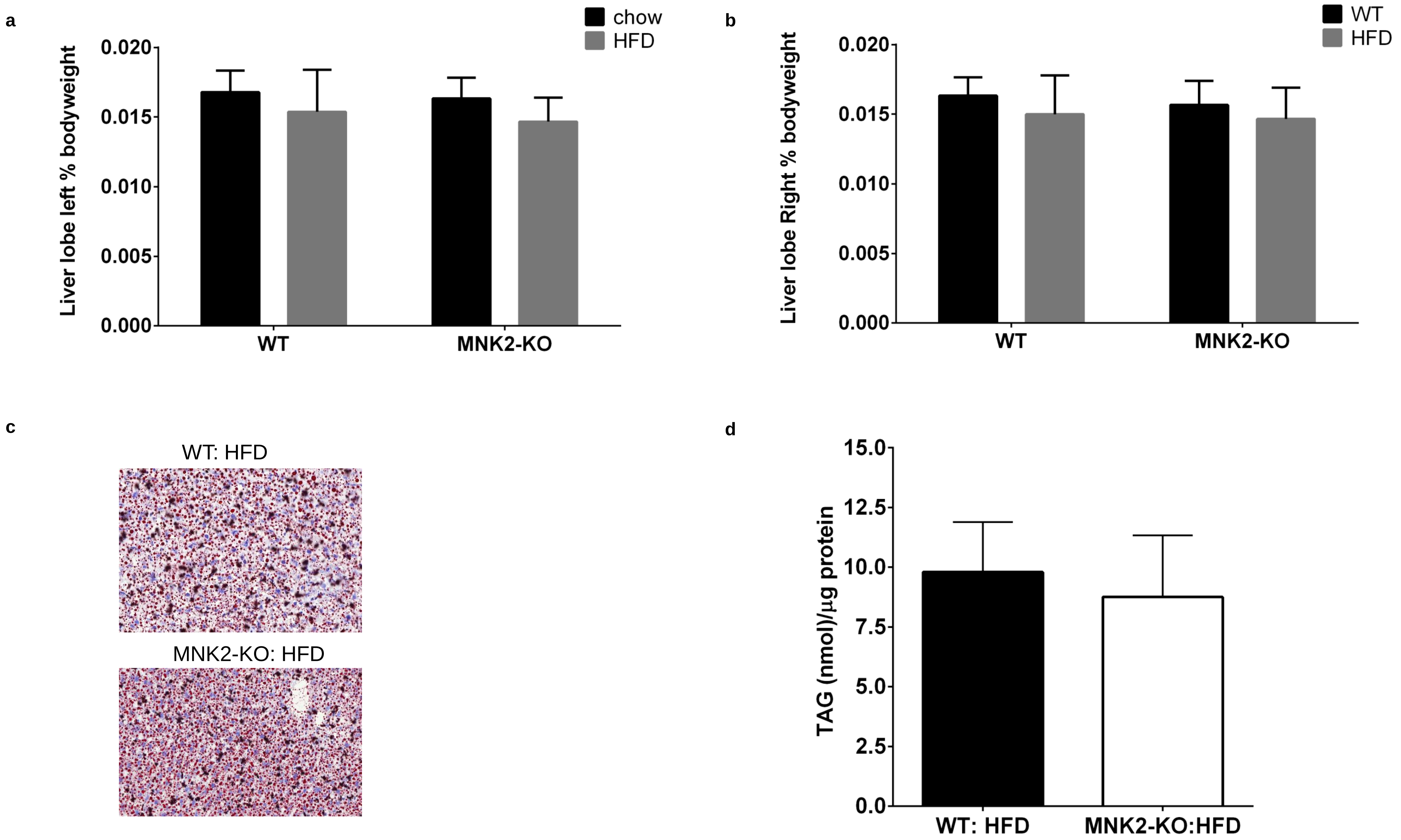

Supplemental Figure 1

Liver histology in WT and MNK2-KO mice (**A,B**) Liver weight of WT and MNK2-KO mice fed chow or the HFD. (n=8-9). Data are mean ± SEM. (**C**) H&E and Oil Red O staining of livers from high fat-fed WT and MNK2-KO mice, 20x magnification. (**D**) Liver total TAG content (n=4-5). Data are mean ± SEM.

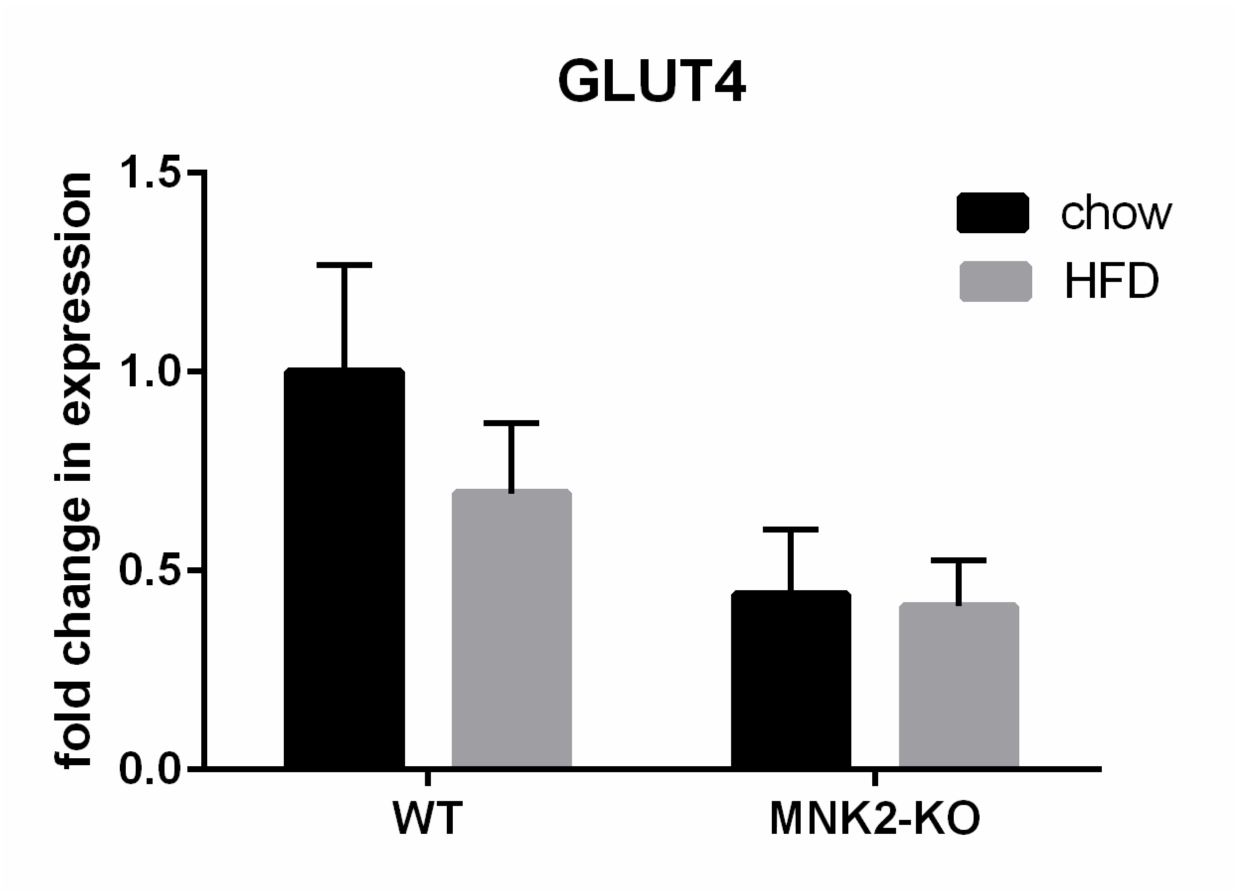

Supplemental Figure 2
Analysis of *GLUT4* mRNA levels in adipose tissue of WT and MNK-2-KO mice (A) Total RNA was isolated from gonadal adipose tissue from chow-fed and HFD-fed WT and MNK2-KO mice. The relative expression of mRNA for *GLUT4* was measured by qPCR (n=4). Data are mean ± SEM relative to WT chow.

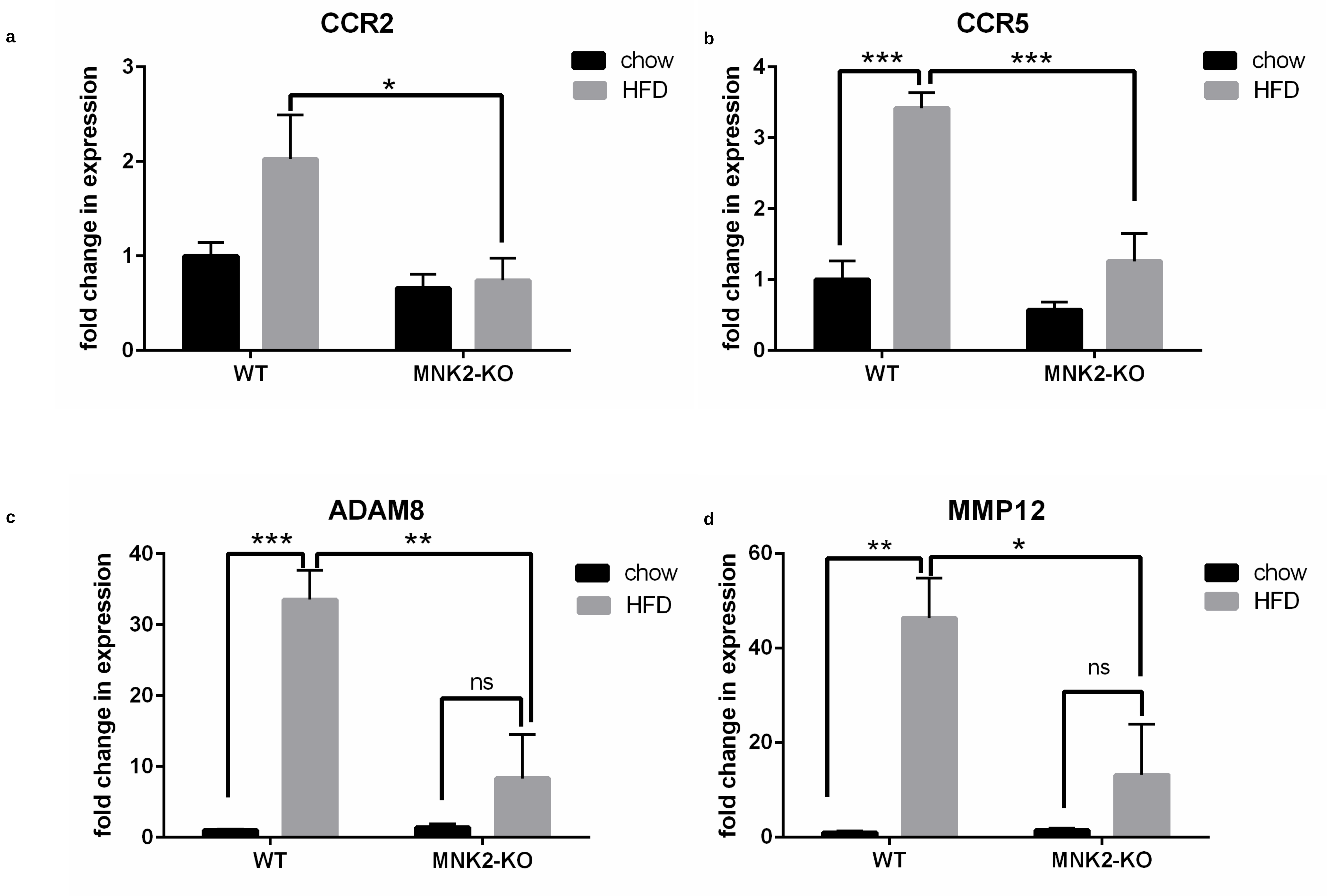

Supplemental Figure 3

Analysis of gene expression in fat tissue from WT and MNK2-KO mice. (**A-D**) Total RNA was isolated from gonadal adipose tissue from chow-fed and HFD-fed WT and MNK2-KO mice. The relative expression of mRNAs for macrophage markers (*CCR2* and *CCR5*) and tissue remodeling markers (*ADAM8 and MMP12*) were measured by qPCR, (n=4). Data are mean ± SEM relative to WT chow (two-way ANOVA followed by Tukey's post test) * P <0.05, ** P<0.01, *** P<0.001.

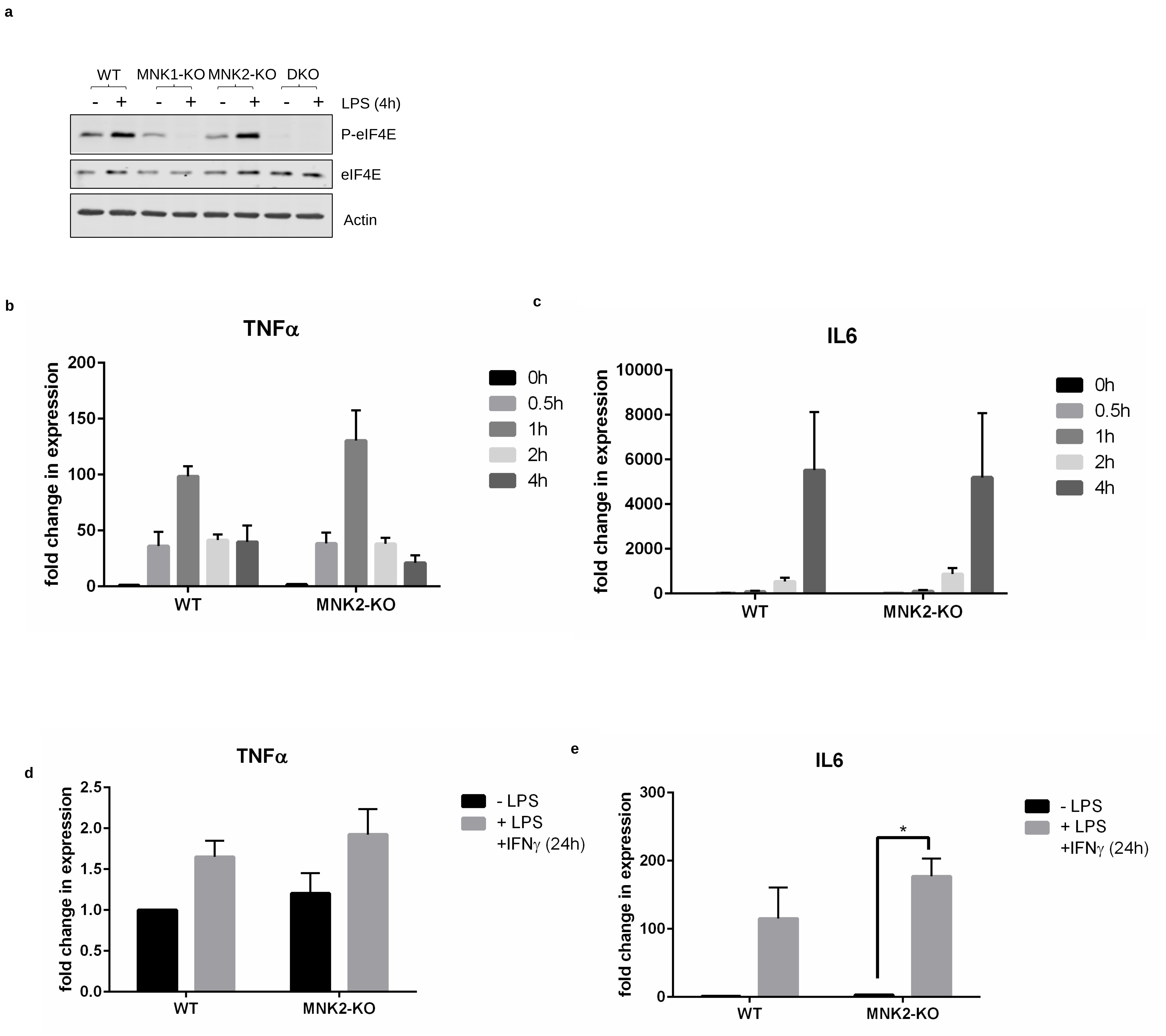

Supplemental Figure 4

Analysis of gene expression in stimulated BMDMs from WT and MNK2-KO mice. (**A**) BMDMs isolated from WT, MNK1-KO, MNK2-KO and MNK1/2-DKO mice were cultured for 4 h in the presence or absence of LPS (250 ng/ml). Lysates were analysed by immunoblot using the indicated antibodies, representative of 3 independent experiments. (**B,C**) BMDMs isolated from WT and MNK2-KO mice were treated with LPS (250 ng/ml) for the times indicated. Total RNA was isolated and the relative expression of mRNAs for *TNFα* and *IL6* was measured by qPCR (n=3). Data are mean ± SEM relative to WT - LPS. (**D**,**E**) BMDMs isolated from WT or MNK2-KO mice were cultured for 24 h in the presence or absence of LPS (100 ng/ml) and IFNγ (20 ng/ml) to polarize the BMDMs towards an M1 phenotype. The mRNA expression levels of the following M1 markers *TNFα* and *IL-6* were measured by qPCR (n=3). Data are mean ± SEM relative to WT - LPS (2-tailed, unpaired Student's *t* test) * P <0.05.

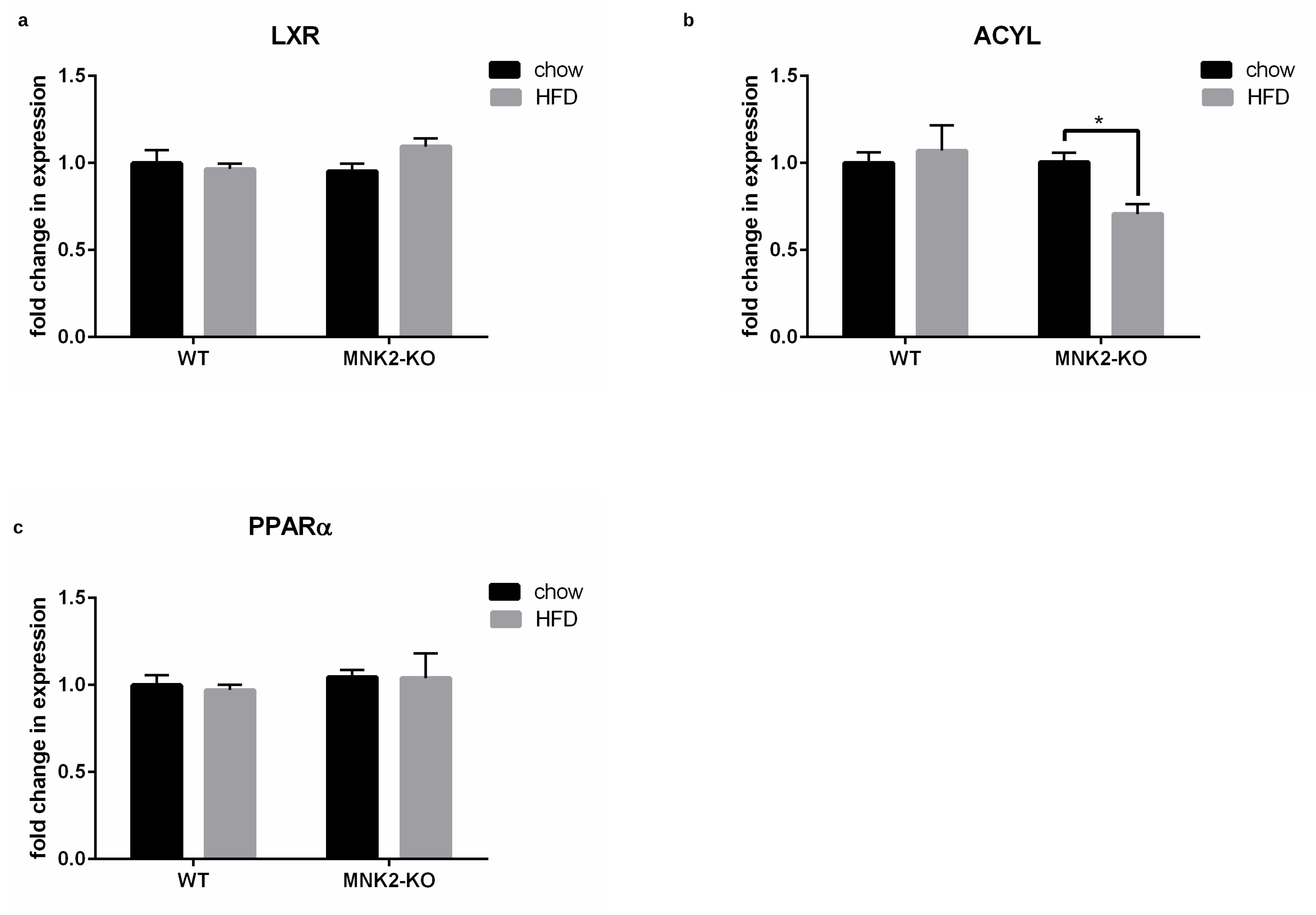

Supplemental Figure 5

Analysis of gene expression in liver from WT and MNK2-KO mice. (**A-C**) Total RNA was isolated from liver tissue from chow fed and HFD fed WT and MNK2-KO mice. The relative expression of *LXR*, A*CYL* and *PPARα* was measured by qPCR (n=4). Data are mean ± SEM relative to WT chow (2-tailed, unpaired Student's *t* test) * P <0.05.

a

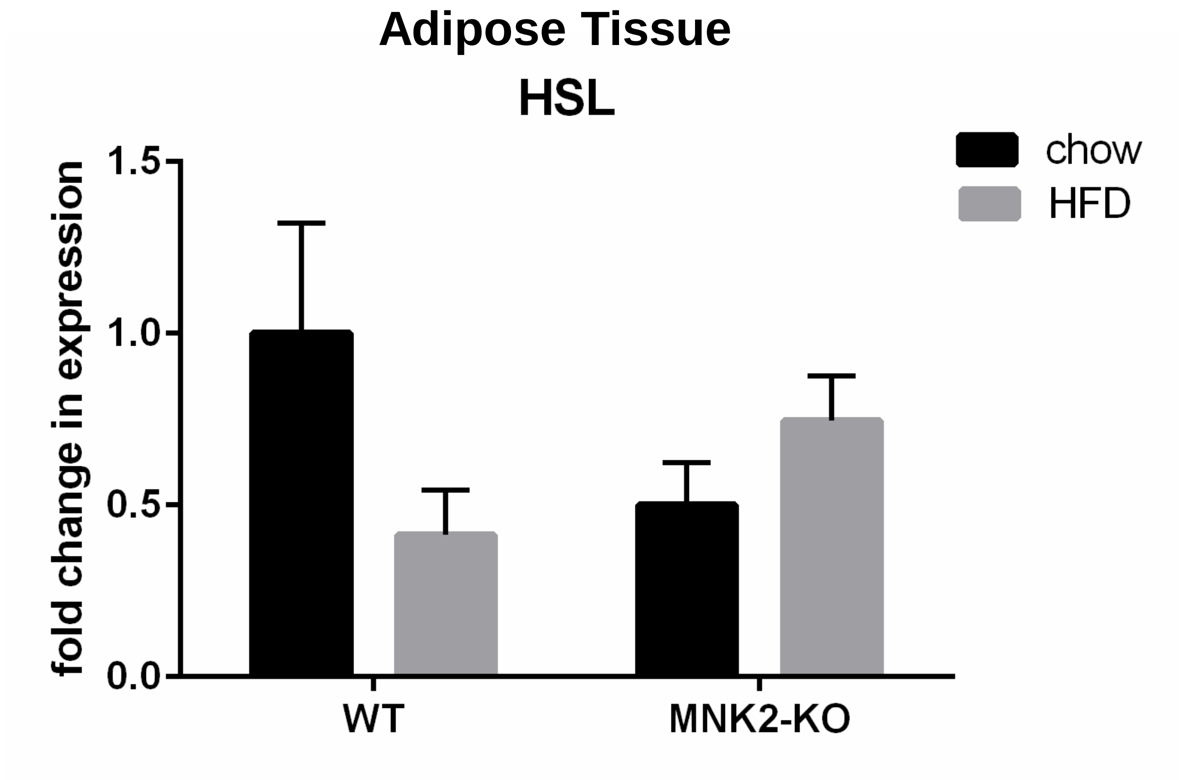

b

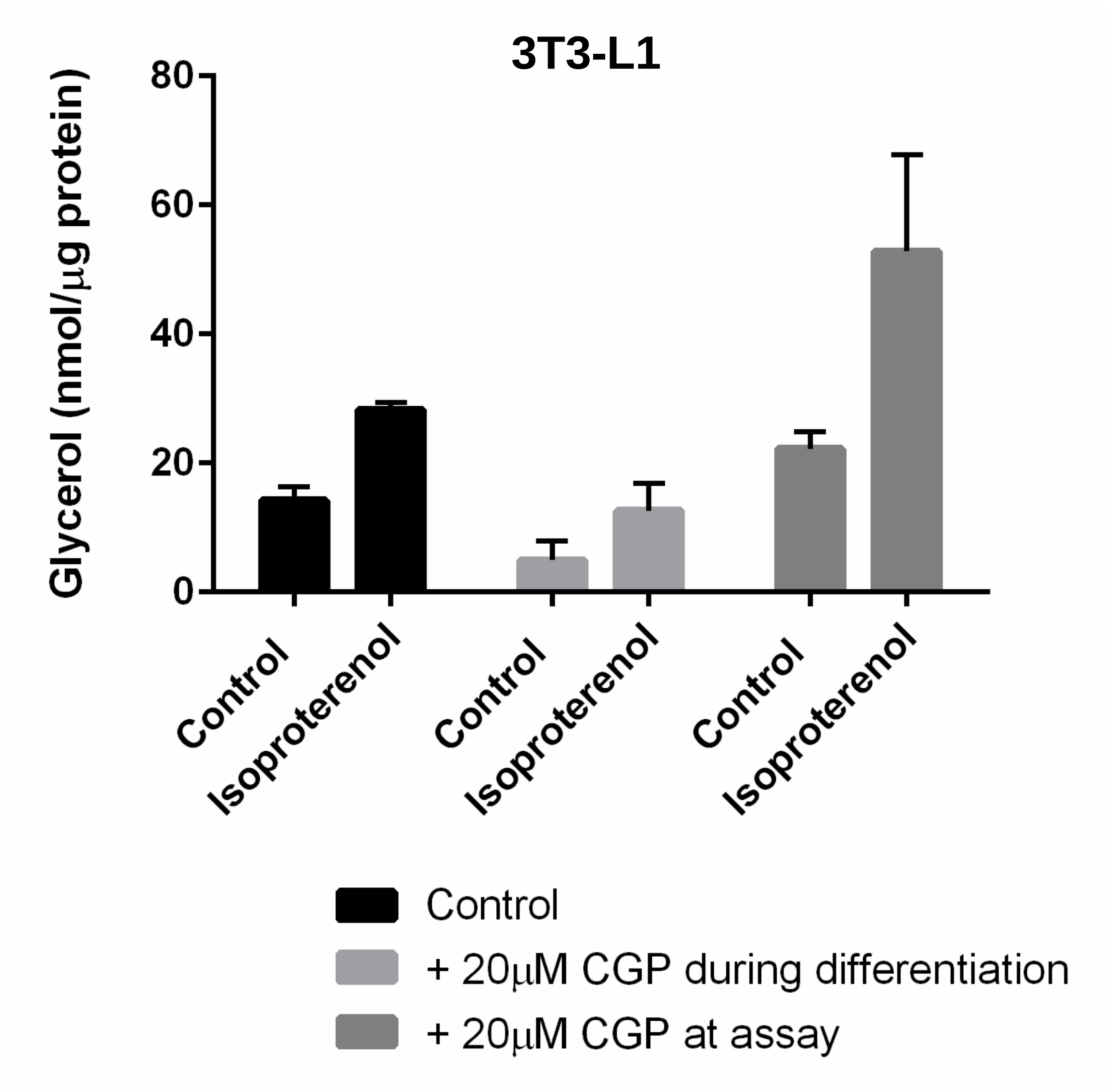

Supplemental Figure 6

Effects of the MNKs on lipolysis. (**A**) Total RNA was isolated from gonadal adipose tissue from chow-fed and HFD-fed WT and MNK2-KO mice. The relative expression of *HSL* was measured by means of qPCR (n=4). Data are mean ± SEM relative to WT chow (**B**) Lipolysis assay: 3T3-L1 cells were differentiated for 9 days in the presence or absence of 20 µM CGP57380. Cells were treated with 100 nM isoproterenol for 3 h to induce glycerol production, in some cases 20 µM CGP57380 was added at the start of the isoproterenol treatment.
